# The influence of family accommodation on pediatric hospital experience in Canada

**DOI:** 10.1186/s12913-017-2529-0

**Published:** 2017-08-15

**Authors:** Linda S. Franck, Deron Ferguson, Sarah Fryda, Nicole Rubin

**Affiliations:** 1 Department of Family Health Care Nursing, University of California, 2 Koret Way, N411Y, Box 0606, San Francisco, CA 94143-0606 USA; 2 ABILITY Network, Inc., 500 Union St., Suite 940, Seattle, WA 98101 USA; 3 National Research Corporation, 1245 Q St., Lincoln, NE 68508 USA; 4 Impact Solutions, 14 Buck Meadow Drive, Portola Valley, CA 94028 USA

**Keywords:** Quality indicators, Patient satisfaction, Patient centered care, Children, Hospital care, Patient and family experience, Family accommodation

## Abstract

**Background:**

The goals of our study were to describe the types of family accommodation for parents of hospitalized children and to examine their influence on the pediatric hospital experience.

**Methods:**

This multi-site cohort survey included 10 hospitals in Ontario Province, Canada. Participants were parents of inpatient children (*n* = 1240). Main outcome measures included ratings of three parent-reported measures of hospital experience: overall hospital experience; willingness to recommend the hospital to family or friends; and how much the accommodation type helped parent stay involved in their child’s hospital care.

**Results:**

Parents most often stayed in the child’s room (74.7%), their own home (12.3%), hotel (4.0%) or a Ronald McDonald House (3.0%). Accommodation varied based on hospital, parent and child factors. Length of stay and the child’s health status were significant predictors for overall hospital experience and recommending the hospital to family or friends, but accommodation type was not. Families who stayed at a Ronald McDonald House reported greater involvement in their child’s care compared with other accommodation types (odds ratio: 1.54–20.73 for contrasted accommodation types).

**Conclusion:**

Use of different overnight accommodations for families of hospitalized pediatric patients in Canada is similar to a previous report of U.S. family hospital accommodations. In contrast to the previous U.S. findings, Canadian hospital experience scores were lower and accommodation type was not a significant predictor of overall hospital experience or willingness to recommend the hospital. In Canada, as in the U.S., families who stayed at a Ronald McDonald House reported that this accommodation type significantly improved their ability to be involved in their child’s care.

## Background

A family-centered approach to the pediatric care has been advocated for decades and has become the standard of care in most hospitals [1–3]. The quality of the family hospital experience has only recently been incorporated in health care quality metrics [4–7], and the latest research indicates that better patient and family experience is associated with more favorable clinical outcomes [8]. However, the proximity and support services provided to families to enable involvement is an important dimension of the patient and family hospital experience that has been largely absent in the literature. Two United States (U.S.) studies have examined the effects on patient experience of overnight accommodations for families of hospitalized children [9,10]. These studies suggest that purpose-built accommodation such as Ronald McDonald House® (RMH), which provides lodging and volunteer support services for parents with hospitalized children in a communal setting close to pediatric hospital services, positively influence the family’s sense of togetherness and involvement in their child’s recovery [9]. The research also suggests that the type of overnight accommodation predicts positive hospital experience outcomes and willingness to recommend the hospital to others [10]. We found no published research on family accommodation and hospital experience from any other country. Because of the potential impact of family accommodation on involvement in the child’s care and the overall patient experience, further research is needed across different health systems.

In Canada, as in other predominantly English-speaking countries, patients and family member surveys about their health care experience are widely used to inform health services and policy [11–16]. Survey instruments presently in use, however, do not include questions about family accommodation during their child’s hospitalization. Therefore, an important dimension of the health care experience – the family proximity – cannot be assessed. For this multi-site cohort survey, we added additional questions to a standard Canadian pediatric patient experience survey to measure the use of various accommodation options, and to investigate the influence of type of family accommodation on parent perceptions of the pediatric hospital experience in a national health care delivery system. Our specific research questions were: 1. Where do parents and other family members stay overnight when their child is hospitalized? 2. Does use of the accommodation types by families differ based on patient or family characteristics? 3. Does accommodation type influence overall family experience, willingness to recommend the hospital to a friend, or the perceived role of accommodation in enabling families be involved in their child’s care, after adjusting for available patient, family and hospital characteristics?

## Methods

### Survey design and data collection

Hospitals in Ontario Province were invited to participate in the research if they were a National Research Corporation (NRC) Picker Canada Inpatient Pediatric Patient Experience Survey client during 2013–2014. Hospitals allowed de-identified aggregated data sharing with the study team for the purposes of this project. Hospitals with an average response volume greater than 10 surveys per month were purposively invited in order to include a mix of hospitals with and without an affiliated RMH, hospitals using English and French versions of the surveys, as well as free-standing children’s hospitals and general community hospitals.

Data collection procedures were the same as for the U.S. survey and are reported elsewhere [10]. Briefly, two questions were inserted into the 83-item Inpatient Pediatric Patient Experiences Survey. The surveys are available for review from NRC (nationalresearch.com). The first inserted question asked about the family’s *primary* overnight accommodation during their child’s hospitalization and listed eight response options: Your own home; Home of relatives or friends; Hotel or motel; Room provided by Ronald McDonald House; Room provided by other charitable organization; Separate room provided by hospital; Your child’s hospital room or nearby visiting/waiting area; or Other accommodations. The second inserted question asked the respondent to rate the helpfulness of the family’s primary overnight accommodations with regard to staying involved in their child’s hospital care. The 11-point (0 to 10) response scale chosen for the second question was consistent with other Canadian Patient Experiences Survey—Inpatient Care (CPES-IC) survey questions [11] as well as Hospital Consumer Assessment of Healthcare Providers and Systems (HCAHPS) surveys, which are mandatory for many hospitals in the U.S. [12].

### Sample selection

There were 21 Ontario Province hospitals with pediatric services using the same Inpatient Pediatric Patient Experiences Survey version and that met the inclusion criteria of an average survey response volume of over 10 surveys per month. Ten of these hospitals agreed to participate. The most common reasons for declining participation were institutional concerns about data sharing. Two of the 10 participating hospitals were children’s hospitals, and both of the hospitals had an affiliated RMH. The 8 general hospitals had substantial pediatric patient populations but only 2 had an affiliated RMH. All participating hospitals were located within metropolitan areas. There were no significant differences between the 10 hospitals that participated in the study and the 11 that did not participate with respect to hospital type characteristics (children’s or general; metropolitan or rural, teaching or non-teaching), or affiliation with an RMH. Participating hospitals varied in size between 133 and 597 beds, with an average size of 371 beds overall, compared to an average of 351 beds for the 11 non-participating hospitals (*p* = 0.20).

### Survey procedures

The survey procedures were similar to those used in the U.S. study [10]. The survey was mailed to the parent (or guardian) within 2 weeks of child’s discharge and if a response was not received in 4 weeks, a second survey was mailed. Survey administration was continuous throughout the year, with the number of surveys mailed to patients adjusted each month according to historical response rates and targets set by each hospital. The beginning dates of participation were the same for each hospital, with the beginning point on May 10, 2013. This research was deemed exempt from formal review by the University of California, San Francisco, Committee on Human Research.

### Data analysis

Descriptive statistics were examined for all variables. Four of the 8 accommodation types: child’s room, own home, hotel or motel, and RMH, together accounted for almost 94% of non-missing responses, with the remaining accommodation types each comprising less than 3.0% of responses. For analysis, the responses for staying in the home of a relative or friend (2.0%) were combined with those for “own home” because in both types parents had readily accessible social support in addition to their lodging. The “Other” category included “Hotel or Motel” (4.0%) “Room provided by other charity” (0.3%), “Separate room provided by hospital” (2.9%), and “Other accommodations” (0.9%). Once combined, the “Other accommodation” category comprised 8.1% of responses. Some respondents did not answer the accommodation type question (9.3%), however, this missing rate was not inordinately high compared to other questions on the survey nor determined to be systematic in any other respects.

Patient age was categorized into the following 4 age groups: < 1 year, 1–6, 7–12, and 13+. Child length of stay was categorized as 1–2 days, 3–5, 6–10, and more than 10 days. The two highest length of stay categories (11–21 days and >21 days) were combined to permit an adequate number of subjects for all accommodation types in regression analyses. Hospital type was a dichotomous indicator distinguishing between children’s (*n* = 2) and general acute care hospitals (*n* = 8).

Travel distance for families was measured by the distance in miles between the geocoded location of the hospital and the geocoded postal code centroid of the family’s mailing address used for the survey. Travel distance was collapsed into the following 4 categories: 0–25, 26–50, 51–100, and more than 100 miles. Travel distance was transformed to a log base 2 value and used as a continuous variable in multivariate analyses. Travel distances of less than one mile were given a value of zero on the log-transformed scale.

In all analyses of effects on hospital experience, we used the two global measures of experience, “overall rating” and “would recommend,” and our added custom question on “accommodation helpfulness.” We applied a scoring method used for the HCAHPS survey in the U.S., in which responses were dichotomized into positive or negative scores (e.g., 1 versus 0) [10]. Positive responses for the five-point “overall rating” item included the top two positive responses (i.e., “Excellent” and “Very good”), and for the three-point “would recommend” item included only the most positive option (i.e., “Yes, definitely”). For the 11-point “accommodation helpfulness” item, positive responses included only the two highest responses (i.e., 9 and 10). For each of these measures, results were reported as the percentage of positive responses. We compared unadjusted positive score differences for the two global measures, overall rating and recommendation, and for the helpfulness of accommodation in patient care involvement across accommodation types using chi-square tests of independence.

We used logistic regression to estimate the effect of accommodation type on hospital experience, adjusting for patient, family, and hospital covariates. We estimated three models, with dependent variables as “overall rating,” “would recommend,” and “accommodation helpfulness,” all scored using the dichotomization method described above. In measuring the effect of accommodation type on each of the three experience measures, “Home” was used as the reference category. While it may not be intuitive to consider the concept of “accommodation helpfulness” in relation to the referent category, “Home,” the survey asks all subjects to rate their accommodations, including those staying at home, using the same criteria. The design presupposes “home” as the most familiar and supportive environment. Many would consider having to stay in a different environment as an additional stress to their child’s hospitalization; we attempted to test that assumption.

The models included all child covariates (gender, age group, overall health rating, and length of stay), the parents’ distance traveled and indicators for each hospital to adjust for hospital-related effects. The accommodation type variable was a categorical indicator using the collapsed accommodation categories described above for Home, RMH, Child’s room, or Other. The hospital type indicator (children’s or general) was not included in the models due to its correlation with the hospital indicators and accommodation types; we assume that the individual hospital indicators adequately capture site-specific effects, including type of hospital. Pairwise adjusted odds ratios were calculated for each accommodation type compared with the others for the relative likelihood of parents reporting a positive score on each of the experience measures. Data analysis was conducted using SAS® v. 9.2 (Cary, NC). A *p*-value of 0.05 was considered statistically significant.

## Results

There were 1390 surveys returned from parents/guardians of children discharged from the hospitals, of which 37 were excluded because of missing data for accommodation type and 90 cases excluded because of spurious entries. Additionally, 23 responses from parents/guardians of neonatal intensive care unit patients were excluded due to the low sample size for that group. The final sample included 1240 responses for pediatric inpatients. Approximately 17% of returned surveys were completed in French, while 83% were completed in English. The response rate for the survey overall was 28%, varying from 18 to 31% across hospitals.

### Families’ use of accommodations during a child’s hospitalization

The most frequent parent overnight accommodation type was the child’s hospital room (74.7%), followed by their own home or that of a relative or friend (14.3%), hotel (4.0%), RMH (3.0%), another room provided by the hospital (2.9%) or other unspecified accommodation (0.9%). Only distance travelled, length of stay, and affiliation with an RMH differed across the accommodation types (Table 1), which is consistent with the purpose of the RMH program to prioritize families traveling the furthest distances and expecting the longest stays away from home. Several factors that varied significantly with accommodation type in the similar U.S. study [10] were not found to vary significantly in this study, including child age, child overall health, and the children’s hospital indicator.

**Table 1:** Comparison of patient, family, and hospital characteristics by accommodation type

Variable	Home/ Relative/ Friend %^a^ *n* = 176	RMH %^a^ *n* = 36	Child’s room %^a^ *n* = 915	Other (incl. Separate hospital room, hotel) %^a^ *n* = 98	*p* value
Total sample: *N* = 1240	14.3	3.0	74.7	8.1	
Child gender^b^					0.638
Male	15.3	3.2	73.7	7.8	
Female	13.1	2.7	75.8	8.4	
Child age (years)^c^					0.483
< 1	13.8	3.7	75.1	7.4	
1–6	15.1	3.9	73.4	7.6	
7–12	11.0	2.5	76.8	9.6	
13 or older	16.9	2.2	73.5	7.4	
Child overall health^d^, *M (SD) (1 = excellent to 5 = poor)*	2.18 (1.09)	2.28 (1.03)	2.12 (1.03)	1.98 (.94)	0.682
Distance travelled (miles)^c^					<.001
0–25	17.0	0.2	74.7	8.0	
26–50	10.7	3.4	81.9	4.0	
51–100	3.2	12.8	78.7	5.3	
> 100	4.2	22.5	50.7	22.5	
Length of stay (days)^c^					<.001
1–2	14.3	1.5	77.6	6.6	
3–5	12.7	4.2	74.6	8.5	
6–10	13.0	6.2	75.3	5.5	
11–21	10.7	3.6	71.4	14.3	
>21	8.7	21.7	65.2	4.3	
Children’s hospital^b^					0.829
No	14.4	2.7	75.4	7.6	
Yes	14.2	3.3	73.9	8.6	
Affiliation with RMH Chapter^b^					<.001
No	17.8	0.0	71.6	10.6	
Yes	12.5	4.5	76.2	6.8	

*RMH* Ronald McDonald House

^a^Percentage of total responses for each item

^b^Chi-square test

^c^Kruskal-Wallis test

^d^One-way analysis of variance

### Variation of hospital experience scores across types of accommodation

Of the three outcome measures, only accommodation helpfulness differed significantly based on accommodation type. For each outcome, families using the RMH reported nominally higher positive scores than the three other types of accommodation, with the highest (and only significant) differential observed for the helpfulness of accommodation (Table 2).

**Table 2:** Comparison of positive global experience scores by accommodation type

Global experience measures	Home/ Relative/ Friend %	RMH %	Child’s room %	Other (including separate hospital room, hotel) %	*p* value^c^
Hospital experience^a^	79.0	89.2	85.2	86.0	0.156
Would recommend to friends and family^b^	79.9	89.2	83.8	82.8	0.465
Accommodation helped maintain involvement in child’s care^b^	60.8	91.9	69.6	64.6	0.002

Note. *RMH* Ronald McDonald House

^a^Percent reporting top two responses

^b^Percent reporting positive score

^c^Chi-square test

### Effect of accommodation type on hospital experience

**Fig. 1:**
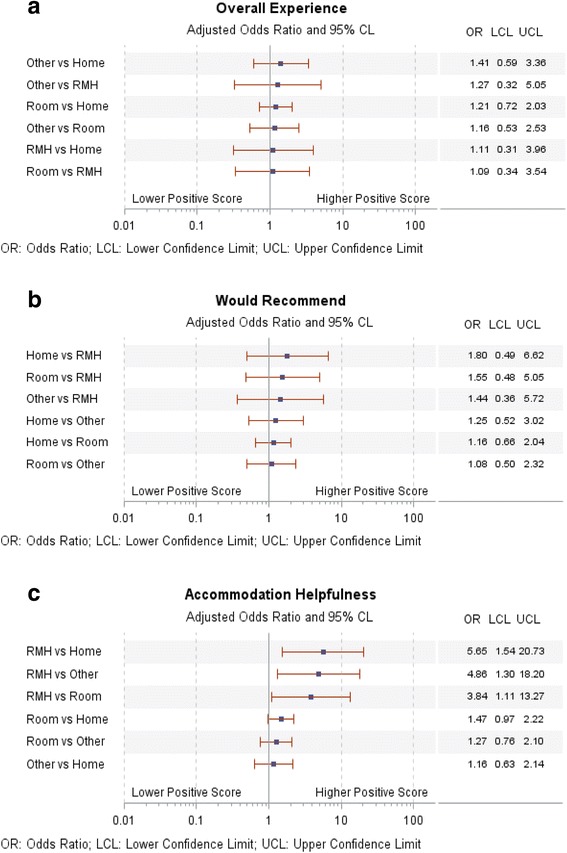
Panels **a** to **c**. Pairwise odds of higher positive scores for family accommodation types. Note. *RMH* Ronald McDonald House. Odds ratio adjusted for patient age and gender, length of stay, child health rating, and hospital type

Models explaining the likelihood of a positive report from families for the global experience measures were significant (likelihood ratio tests: *p* < .05 to <0.001). Accommodation type was not a significant predictor for the two overall outcomes (overall rating and willingness to recommend). The covariate-adjusted odds ratios comparing each accommodation type with the others are shown in Fig. 1, Panel a, for overall hospital experience and in Fig. 1, Panel b, for willingness to recommend the hospital to friends or family. None of these differences were significant.

Positive ratings of the helpfulness of accommodation in parents’ involvement in their child’s care (measured using the 0–10 response scale) were significantly higher for families staying in RMH (OR home: 5.65, 1.54–20.73; other: 4.86, 1.30–18.20; room: 3.84, 111–13.27) (Fig. 1, Panel c). Comparisons between RMH and other accommodation types were all significantly in favor of RMH with respect to helpfulness of accommodation.

Important (significant) adjustment covariates included length of stay and parent rating of child health (Table 3). A lower child’s health rating (modeled as a continuous variable) uniformly reduced the likelihood of positive experience scores in each of the models. For each 1- point increase in the child health overall rating score, the odds of a positive response for overall experience declines by about 31%, and for would recommend and accommodation helpfulness the odds decline by 35% and 21%, respectively. There was significant variation in the odds of positive scores based on the hospital (not shown).

**Table 3:** Multivariate logistic regression analysis of global experience measures

	Adjusted odds ratios [95% CI]					
Covariate^a^	Overall experience		Would recommend		Accommodation helpfulness	
Accommodation type (ref = home)						
RMH	1.11	(0.31–3.95)	0.56	(0.15–2.04)	5.65	(1.54–20.73)^b^
Room	1.21	(0.72–2.03)	0.86	(0.49–1.52)	1.47	(0.97–2.22)
Other	1.41	(0.59–3.36)	0.80	(0.33–1.92)	1.16	(0.63–2.14)
Distance (log 2)	1.02	(0.91–1.14)	1.10	(0.98–1.24)	1.03	(0.96–1.12)
Length of stay (ref = 1–2 days)						
3–5 days	1.43	(0.90–2.25)	1.11	(0.70–1.76)	0.93	(0.67–1.29)
6–10 days	1.21	(0.67–2.16)	1.31	(0.69–2.50)	0.91	(0.59–1.38)
11 or more days	1.46	(0.64–3.32)	2.10	(0.60–7.28)	1.01	(0.52–1.94)
Patient’s age group (ref ≤ 1 year)						
1–6 years	0.86	(0.47–1.52)	0.96	(0.55–1.70)	1.34	(0.86–2.09)
7–12 years	0.91	(0.49–1.65)	0.90	(0.50–1.63)	1.03	(0.66–1.59)
13 or more years	0.67	(0.35–1.25)	0.57	(0.30–1.08)	1.19	(0.74–1.89)
Patient’s gender (ref = female)	0.72	(0.49–1.05)	0.85	(0.58–1.26)	1.12	(0.85–1.47)
Child’s health rating (1 = *excellent* to 5 = *poor)*	0.59	(0.49–0.69)^b^	0.65	(0.54–0.78)^b^	0.79	(0.69–0.90)^b^

Note. *RMH* Ronald McDonald House

^a^Facility indicators included in model (estimates not shown)

^b^Model coefficients below .05% significance level

## Discussion

Parent overnight accommodation during a child’s inpatient hospital stay and its relationship to patient and family hospital experience has recently been reported in the U.S. context [10] but until now was not known for Canada. Our findings suggest that parents have similar overnight accommodation options and use them with similar frequency in Canada and the U.S. In contrast to the U.S. study, no significant differences were found between RMH and other accommodation types for overall hospital rating and willingness to recommend the hospital. The Canadian survey instrument used a fewer number of response options for each measure (5-point and 3-point response options for overall rating and willingness to recommend, respectively) whereas the U.S. study used an 11-point scale, providing for greater variation in responses and precision to detect differences. Hospitals across Canada are converting to the new CPES-IC standardized survey to gather patient feedback about the quality of hospital care. The CPES-IC includes 22 items from the HCAHPS [12] survey, 19 questions that address key areas relevant to the Canadian context and 7 questions to collect demographic information [11]. The CPES-IC uses a 0–10 rating scale for the overall rating, which may improve precision. However, the 0–3 scale for the willingness to recommend item remains the same as in the current survey.

Fewer patient, family and hospital characteristics were measured in the Canadian sample compared to the U.S. study and so future studies are needed to determine if there is variation in accommodation type use by Canadian parents for factors other than distance from home, length of stay and RMH affiliation. In contrast to our previous U.S. findings, many fewer families residing more than 100 miles from the hospital were likely to stay overnight in the child’s room [10]. One possible explanation for this may be greater availability of nearby RMH or other accommodation. However, further research is needed to better understand the factors influencing decisions on accommodation availability and choice. The finding of a significant relationship between lower child’s health rating and reduced likelihood of positive experience scores has been reported previously [13]. It is important to understand the relationships between family characteristics and accommodation types for families in different contexts, assure equity in availability of accommodation support, and to address specific conditions that prevent families from being present and actively participating in their child’s hospital care [1–3, 9].

Health care institutions across the world appear to be answering the call to include patient experience as an important measure of health care quality and to provide greater patient and family engagement [14–16]. However, they appear to be neglecting the important role of overnight accommodation and family support services in enabling families to engage in their child’s hospital care. Health care institutions and health systems should incorporate into their patient experience surveys questions about distance between the hospital and the family home and the availability of overnight accommodation, as well as information on the need for child or elder care, employment and transportation constraints. These study findings continue to build the evidence that accommodation is a critical element to hospital efforts to encourage and support family-centered care.

The present findings provide further evidence of the need for questions about family accommodation to be included in all hospital experience surveys so that its influence on hospital experience can continue to be studied. The findings in Canada were overall consistent with the results of a similarly designed study in the U.S. [10]. Differences such as the lack of significant difference in experience scores of families who stayed at an RMH compared to other accommodation types may be due to the smaller sample size, cultural differences around family expectations of the hospital experience, or may be genuine. Further research should compare the effectiveness of strategies to increase access to purpose-built family accommodation and supportive services, such as those provided by the RMH. Research is also needed to investigate the mechanisms by which a supportive family environment proximal to the patient care setting influences hospital experience and quality of care. It would also be meaningful to compare the influence of accommodation on patient experience found in the Canadian and U.S. studies to patient experience ratings in other countries.

Some study limitations are noted. First, the hospital sample was a convenience sample from one Canadian province and may not be representative. Second, the response rate was low, although in a range typical for patient experience surveys [16]. The sample size may not have been large enough to detect significant effects, although a strength in this regard is that, while not statistically significant, many differences among the variables across accommodation types demonstrated similar nominal patterns as the U.S. study [9]. As noted above, there was a lack of precision in some of the outcome response scales and also a limited number of adjustment variables compared to the U.S. study [9]. Proximity of all accommodation opportunities could also be an important factor influencing the choice of accommodation, and thus of the impact on hospital experience. This study could not collect location data for each accommodation in order to address this influence, but further detail on location of accommodation could be a useful addition to future research. Study strengths included its methodology, imbedding the research question in well-established, well-validated ongoing survey procedures, and the diversity of the participating hospitals and respondents.

## Conclusion

In summary, we found that parents’ use of overnight accommodation for hospitalized infants and children in Ontario, Canada varies based on hospital and child level of illness. This study supports the U.S. study findings that there are relationships between parent overnight accommodation type and patient and family hospital experience, although statistically significant effects were not found. In particular, it finds a significant relationship with parents’ perception of support (helpfulness) deriving from the type of accommodation. We strongly recommend that accommodation type be routinely measured in all patient and family experience hospital surveys so that its effects on health care quality and safety can be further examined. We urge public and private hospitals and health systems to consider the importance of family accommodation and support services as an essential component of family-centered care.

## Abbreviations

CPES-IC: Canadian Patient Experiences Survey—Inpatient Care
HCAHPS: Hospital Consumer Assessment of Healthcare Providers and Systems
NRCC: National Research Corporation Canada
RMH: Ronald McDonald House
US: United States
